# Efficient polymer dimerization method based on self-accelerating click reaction†

**DOI:** 10.1039/c9ra09919k

**Published:** 2020-02-13

**Authors:** Xueping Liu, Ying Wu, Minghui Zhang, Ke Zhang

**Affiliations:** State Key Laboratory of Polymer Physics and Chemistry, Institute of Chemistry, The Chinese Academy of Sciences Beijing 100190 China kzhang@iccas.ac.cn; Institute of Polymer Chemistry and Physics, Beijing Key Laboratory of Energy Conversion and Storage Materials, College of Chemistry, Beijing Normal University Beijing 100875 China; School of Chemistry and Chemical Engineering, University of Chinese Academy of Sciences Beijing 100049 China

## Abstract

An efficient polymer dimerization method is developed on a self-accelerating double strain-promoted azide–alkyne cycloaddition (DSPAAC) click reaction. In this approach, varied polymer dimers can be efficiently prepared by coupling azide terminated polymer building blocks by *sym*-dibenzo-1,5-cyclooctadiene-3,7-diyne (DIBOD) small linkers. The distinct advantages of this method can be summarized as follows. First, the azide terminated polymer building blocks can be easily prepared with varied molecular topologies such as linear, star, and dendritic shapes. Second, the self-accelerating property of DSPAAC coupling reaction allows the method to efficiently prepare pure polymer dimers in the presence of excess molar amounts of DIBOD small linkers to azide-terminated polymer building blocks. Third, the click property of DSPAAC coupling reaction facilitates the dimerization reaction with a very mild ambient reaction condition. As a result, this method provides a powerful tool to fabricate topological polymers with a symmetrical molecular structure such as block, star, and dendritic polymers.

## Abstract 2

A convenient and efficient method was developed to prepare topological polymers with a symmetric molecular structure by dimerizing azide terminated polymers based on the self-accelerating double strain-promoted azide–alkyne cycloaddition reaction.

## Graphical abstract

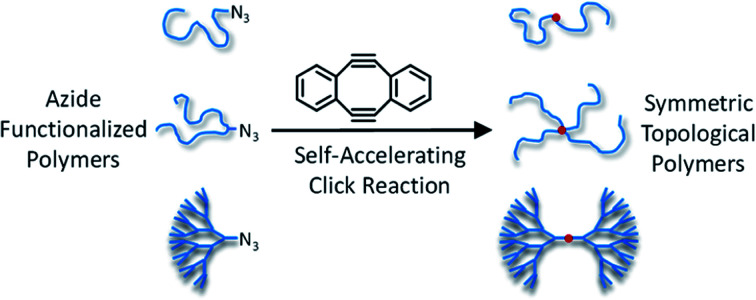

## Introduction

In nature, the dimerization of biomolecules is a phenomenon worthy of researching, such as the ultraviolet induced thymine dimerization in DNA,^1^ the dimerization of transmembrane receptors in cellular communication,^2^ and the dimerization of HIV-1 protease.^3^ In the field of synthetic polymers, the dimerization of polymer chains produced a topologically symmetric dimer of double molecular weight compared to the precursor. As an example, Tillman's group achieved the dimerization of poly(methyl methacrylate) (PMMA) chains using radical trap-assisted atom transfer radical coupling reaction, which altered the mechanistic pathway of the traditional radical–radical termination of PMMA.^4^ Using 1,3-diazidopropane as the small molecular linkers, Wang *et al.* prepared the dimers of dibenzocyclooctyne-terminated polystyrene (PS) and poly(ethylene oxide) (PEO) *via* strain-promoted azide–alkyne cycloaddition (SPAAC), respectively.^5^ More importantly, the dimerization of nonlinear polymers provided an efficient synthetic approach for symmetric polymers with complex topologies. As early as 1998, Kubo *et al.* prepared the 8-shaped PS *via* dimerization of cyclic PS with an amide moiety, using glutaric acid as the linkers.^6^ Tezuka *et al.* reported the dimerization of cyclic poly(tetrahydrofuran) (poly(THF)) with an allyl group *via* the intermolecular metathesis condensation in the presence of a Grubbs catalyst, to produce the 8-shaped poly(THF).^7^ Lee's group reported a series of research studies on the dimerization of dendrons (both Fréchet-type polyether and Tomalia-type PAMAM) to produce cycloaddition (CuAAC) or Glaser coupling reactions.^8–11^ Dimerization is indeed effective for the construction of symmetric polymer systems, however, the reported methods suffer from at least one of the following drawbacks: (1) the coupling reactions (such as metathesis condensation, CuAAC and Glaser reactions) require the metal catalysts, which become impurities and difficult to be removed completely. (2) The dimerization using small molecular linkers requires accurate 2 : 1 molar ratio between the polymers and the difunctional linkers, in order to guarantee the purity of the resultant dimers. In practice, the accurate 2 : 1 molar ratio is hard to achieve due to the molecular weight distribution of the polymers. (3) The purification of the products become indispensable because of the impurities mentioned above. The commonly used methods are silica gel column chromatography and preparative size-exclusion chromatography, which are clearly not qualified as simple and convenient purification methods.

Since introduced by Sharpless in 2001, the click chemistry has been widely used as a powerful tool in the fields of organic chemistry, polymer chemistry, biology, and material science.^12–20^ To date, several click reactions have been developed including copper(i)-catalyzed azide–alkyne cycloaddition (CuAAC),^21^ Diels–Alder reactions,^22^ thiol–ene coupling,^23^ nucleophilic substitution of activated esters with amines,^24^ and multicomponent reactions.^25^ Among them, the multicomponent click reactions have gained intense attention to construct functional polymer materials due to their “one-pot” and atom economy reaction properties.^26–28^ Recently, Barner-Kowollik and colleagues reaffirmed the core ideas of click chemistry with an adapted definition in the context of polymer chemistry, in order to clearly classify the click reactions.^29^ Briefly, the following criteria are required: (1) click reactions are intrinsically highly efficient reactions (but not all efficient reactions are classified as click reactions); (2) the product separation is easy operating (by nonchromatographic methods, such as crystallization or distillation); (3) the click reactions are performed under equimolar conditions (as being spring-loaded for a single trajectory).^12^ Especially in the most challenging polymer–polymer conjugation, the usage equal molar amount of complementary plays a key role to guarantee the purity of the product. Selective precipitation can easily remove the excess feeding of one polymer reagent in the purification of asymmetric polymer–polymer conjugates. However, it is difficult to purify a dimerization system with unequimolar amounts of the starting reagents, since the physical properties are similar for both the starting polymer and its dimer. The only difference of double molecular weight leads to the chromatographic methods for separation.

**Scheme 1:**
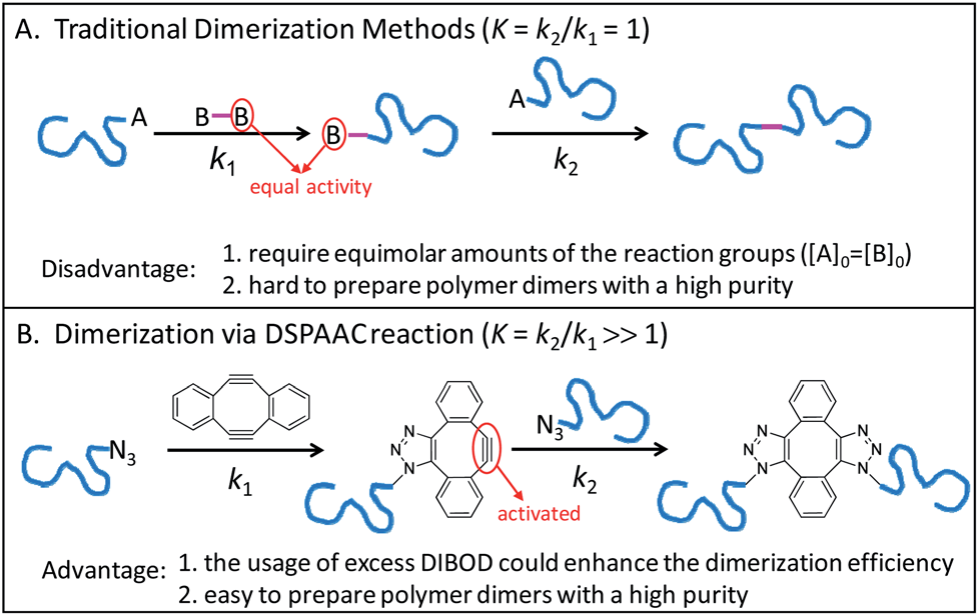
Mechanistic comparison of the traditional dimerization methods (A) to the dimerization *via* DSPAAC reaction (B), where one isomer was used to demonstrated the molecular structure of polymer dimer in case B.

Conjugation of end-functionalized polymer by a difunctional small linker is a common method for polymer dimerization, which follows a two-step reaction process. As shown in Scheme 1A, the reaction of polymer end A group with one B group of small linker produces the polymer with B end group. The polymer dimers are then formed by a subsequent conjugation of polymers with A and B end groups. Since the traditional polymer dimerization methods used the same reactions to perform the two-step couplings, the usage of equal molar amounts of A and B reactive groups was a prerequisite for preparing polymer dimers with a high purity (Scheme 1A). Recently, we introduced a double strain-promoted azide–alkyne cycloaddition (DSPAAC) reaction to prepare the cyclic polymers *via* bimolecular homodifunctional method, using *sym*-dibenzo-1,5-cyclooctadiene-3,7-diyne (DIBOD) as the small difunctional linker.^30–32^ The distinct advantage of the DSPAAC reaction lies in its self-accelerating property, comparing to the traditional azide–alkyne click reactions. As shown in Scheme 1B, the reaction of DIBOD with one azide functionalized polymer chain *in situ* activated the second alkynyl group of DIBOD. The accelerated second step reaction with another polymer chain shows a much higher rate (*k*_2_) than that of the first step (*k*_1_). Resultantly, the self-accelerating DSPAAC reaction is well-suited for the dimerization of polymer chains since it can break through the restriction of equimolar feeding. As a matter of fact, the usage of excess DIBOD could significantly increase the reaction rate of the intermolecular coupling (*k*_1_), and resultantly enhance the efficiency of the dimerization. In addition, different from the well-used CuAAC, the DSPAAC can be performed efficiently under much mild conditions, such as in air, at room temperature, and without requiring any catalysts or chemical stimuli.

**Scheme 2:**
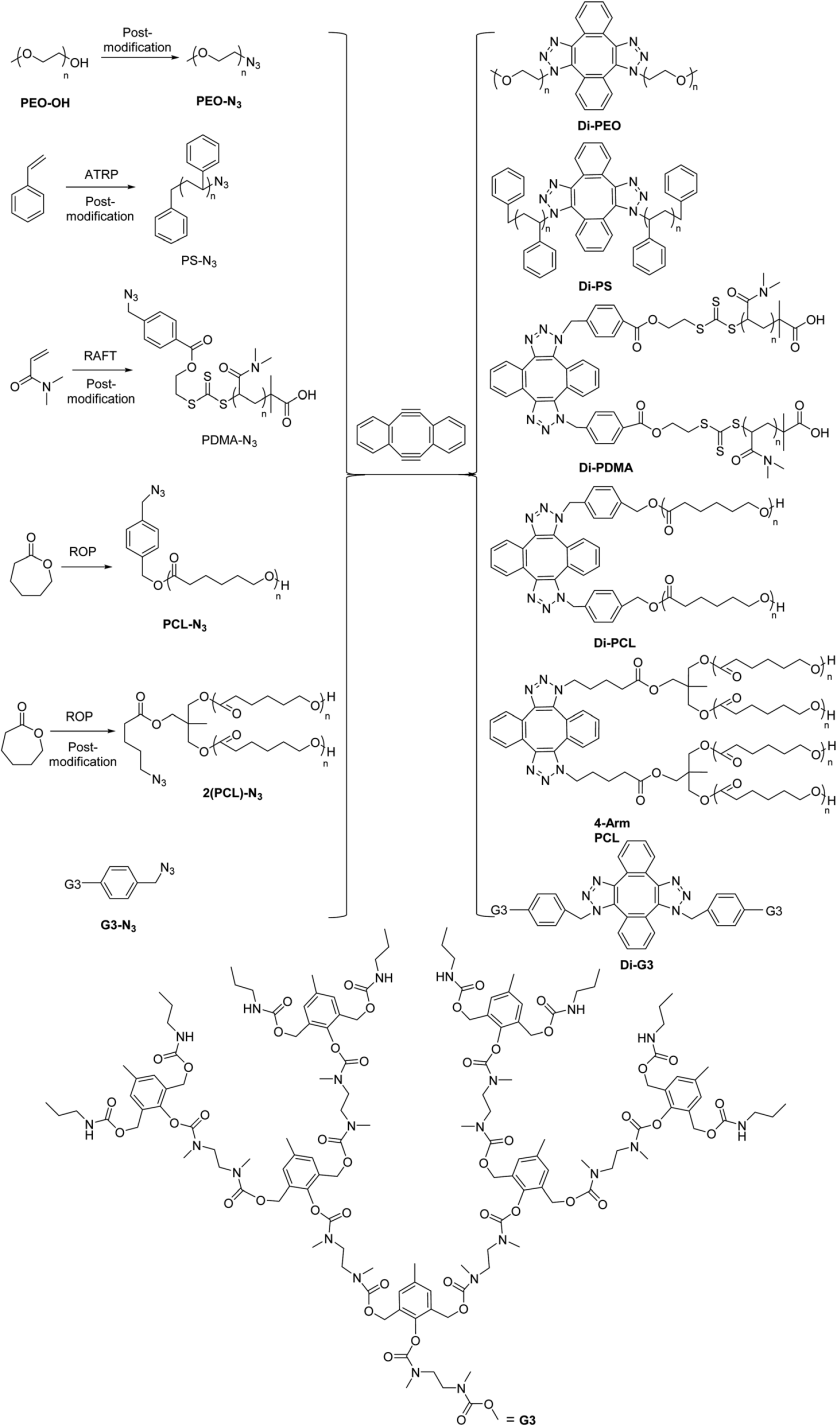
Preparation of polymer dimers based on the combination of post-modification/controlled polymerization and self-accelerating DSPAAC reaction, where one isomer was used to demonstrated the molecular structure of polymer dimers respectively.

Herein, we prepared a series of topologically symmetric polymers by dimerization of azide functionalized polymer blocks, using DIBOD as the small molecular linkers (Scheme 2). The azide functionalized polymers are prepared by either post-modification techniques (*e.g.* PEO-N_3_, azide functionalized third generation dendron G3-N_3_) or controlled polymerization, including PS-N_3_ from atom transfer radical polymerization (ATRP), azide-terminated poly(*N*,*N*-dimethyl acrylamide) (PDMA-N_3_) from reversible addition–fragmentation chain transfer (RAFT) polymerization, azide functionalized poly(ε-caprolactone) (PCL-N_3_ and 2(PCL)-N_3_) from ring-opening polymerization (ROP). Eliminated the requirement of equimolar feeding amounts of the reagents, the respective dimerization of these azide functionalized polymers was performed successfully in the presence of an excess molar amounts of DIBOD, and produced symmetric dimers including linear polymers, 4-arm star polymers and dendrimers.

## Results and discussion

### Dimerization of azide-terminated linear polymers

**Fig. 1:**
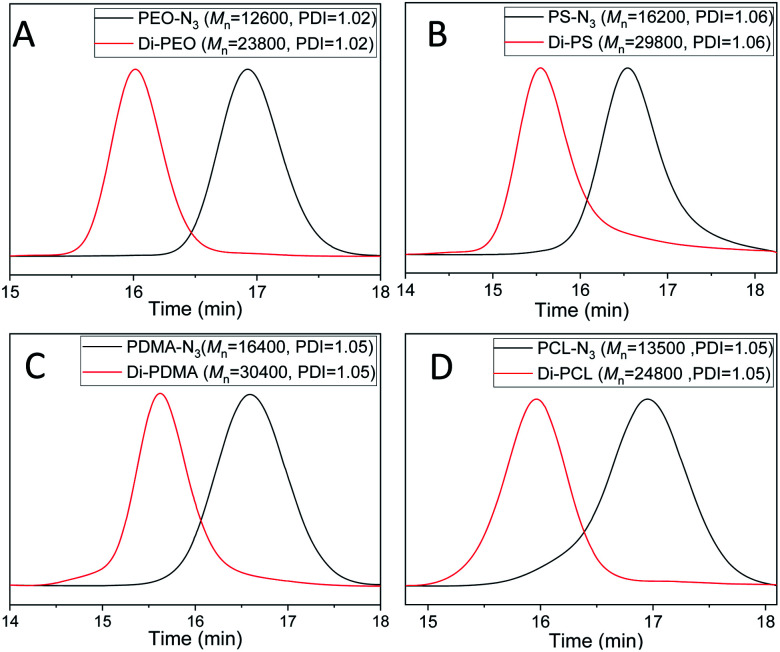
GPC curves of the azide-terminated linear polymers and the resultant dimers: (A) PEO-N_3_ (black) and Di-PEO (red); (B) PS-N_3_ (black) and Di-PS (red); (C) PDMA-N_3_ (black) and Di-PDMA (red); (D) PCL-N_3_ (black) and Di-PCL (red). DMF was used as the eluent, and polystyrene standards were used for calibration.

Post-modification techniques provided an effective way to prepare azide-terminated linear polymers. As an example, linear PEO-N_3_ was prepared by end-functionalizing commercial available PEO-OH, the process of which was detailed in the Experimental section. Briefly, PEO-OH reacted with PTSC first to produce PEO-OTs. In the ^1^H-NMR spectrum of PEO-OTs (Fig. S1B†), the newly formed peaks *H*_b_ and *H*_c_ were ascribed to the *p*-toluenesulfonyl end group, compared to that of PEO-OH (Fig. S1A†). Additionally, the peak area ratio of 2/2/2 among *H*_b_, *H*_c_ and *H*_d_ strongly indicated the quantitative modification. The GPC curves show a monomodal and symmetrical peak shape for both PEO-OTs (Fig. S2A,† red) and its precursor PEO-OH (Fig. S2A,† black). The corresponding *M*_n_ and PDI were 13 400 and 1.02 for PEO-OTs and 13 500 and 1.02 for PEO-OH, respectively. Then the *p*-toluenesulfonyl terminal of PEO-OTs was substituted by azide group in the presence of NaN_3_. From the ^1^H-NMR spectrum of the resultant PEO-N_3_ (Fig. S1C†), the signal of *p*-toluenesulfonyl group disappeared completely. The FT-IR spectrum of PEO-N_3_ (Fig. S3A,† blue) shows the characteristic azide absorption peak at 2100 cm^−1^. Fig. 1A (black) shows the GPC curve of PEO-N_3_, in which a monomodal and symmetrical peak was observed with the corresponding *M*_n_ and PDI of 12 600 and 1.02, respectively. These results strongly demonstrated the successful preparation of PEO-N_3_.

**Fig. 2:**
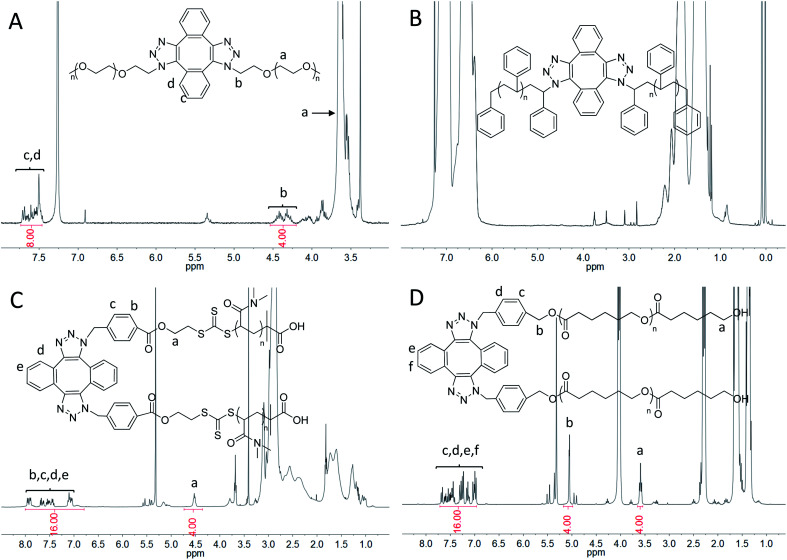
^1^H-NMR spectra of Di-PEO (A), Di-PS (B) in CDCl_3_ and Di-PDMA (C), Di-PCL (D) in CD_2_Cl_2_.

The dimerization of PEO-N_3_ was then performed *via* the self-accelerating DSPAAC. The dimerization efficiency was evaluated under different reaction conditions, including the reaction time and the feed ratio between PEO-N_3_ and DIBOD. The concentration of PEO-N_3_ was fixed at 0.1 mol L^−1^ in a lower boiling point solvent THF. With a molar ratio of PEO-N_3_/DIBOD = 1/1, the GPC curve (Fig. S4A,† red) of the dimerization product shows a bimodal distribution after 5 min reaction. The peak of higher molecular weight was ascribed to the PEO dimer (Di-PEO), and the peak of lower molecular weight was ascribed to the residual PEO-N_3_ (*ca.* 15%). With increasing the reaction time to 10 min, 20 min and 30 min, a monomodal and symmetric peak shape was observed in all cases (Fig. S4A†), indicating a complete dimerization of PEO-N_3_ under these conditions. When the molar ratio of PEO-N_3_/DIBOD decreased to 1/2 and 1/4, the dimerization completed in 5 min, as shown in the GPC characterizations (Fig. S4B and C†). The usage of excess small linker DIBOD could improve the preparation efficiency of the polymer dimers. In this work, a molar ratio of azide-functionalized polymer/DIBOD = 1/1 and the reaction time of 30 min were applied in the dimerization, in consideration of economizing the reagents and ensuring the completeness of the dimerization. From the GPC curve of Di-PEO (Fig. 1A, red), the *M*_n_ was calculated as 23 800, which is approximately double molecular weight of PEO-N_3_ (Fig. 1A, black, *M*_n_ = 12 600). The PDI was calculated as 1.02 for both cases. In the FT-IR spectrum of Di-PEO (Fig. S3B,† magenta), the azide absorption peak disappeared completely at 2100 cm^−1^, indicating a complete consumption of azide terminal of PEO-N_3_. Fig. 2A shows the ^1^H-NMR of Di-PEO, in which the peak area ratio of 4/8 between *H*_b_ (4H of PEO chain) and *H*_c,d_ (8H of DIBOD linker) clearly demonstrated the quantitative formation of Di-PEO.

Azide-terminated linear polymers can also be prepared by controlled polymerization, combined with post-modification process if necessary. For instance, linear PS-N_3_, PDMA-N_3_ and PCL-N_3_ were prepared by ATRP, RAFT and ROP respectively (Scheme 2). The details were included in the Experimental section. In short, PS-Br was prepared by ATRP using benzyl bromide as the initiator. PS-N_3_ was then obtained by reacting PS-Br with NaN_3_. Fig. S2B† shows the GPC curves of PS-Br and PS-N_3_, in which a monomodal and symmetrical peak shape was observed for both cases. The corresponding *M*_n_ and PDI were calculated as 16 300 and 1.06 for PS-Br and 16 200 and 1.06 for PS-N_3_, respectively. In the FT-IR spectra (Fig. S3B†), PS-N_3_ shows the characteristic azide absorption peak at 2100 cm^−1^, compared to that of PS-Br. In the ^1^H-NMR spectra (Fig. S5†), the peak of α-H of the terminal moved from 4.50 ppm (*H*_a1_ of PS-Br) to 3.95 ppm (*H*_a2_ of PS-N_3_) after azidation. For the synthesis of PDMA-N_3_, RAFT polymerization was applied to prepare PDMA-OH first, and then PDMA-OH reacted with 4-(azidomethyl)benzoic anhydride to produce PDMA-N_3_. The GPC curves of both PDMA-OH and PDMA-N_3_ (Fig. S2C†) show a monomodal and symmetrical peak shape, with corresponding *M*_n_ and PDI of 16 500 and 1.05 for PDMA-OH and 16 400 and 1.05 for PDMA-N_3_, respectively. The FT-IR spectrum of PDMA-N_3_ (Fig. S3C,† red) shows the characteristic azide absorption peak at 2100 cm^−1^, compared to that of PDMA-OH (Fig. S3C,† black). In the ^1^H-NMR spectra (Fig. S6†), the newly formed peaks of *H*_c2,d2,e2_ were assigned to the protons of azidomethyl benzoic end group of PDMA-N_3_, compared to that of PDMA-OH. Additionally, the peak area ratio of 1/2/2 among *H*_b2_, *H*_c2_ and *H*_d2_ clearly indicated the quantitative post-modification of PDMA-N_3_. Comparatively, PCL-N_3_ was simply prepared by ROP, using an azide functionalized initiator. Fig. 1D (black) shows the GPC curve of PCL-N_3_, with corresponding *M*_n_ and PDI of 13 500 and 1.05, respectively. The FT-IR spectrum of PCL-N_3_ (Fig. S3D,† black) shows the characteristic azide absorption peak at 2100 cm^−1^. In the ^1^H-NMR spectrum of PCL-N_3_ (Fig. S7†), the peak area ratio of 2/2 between *H*_a_ and *H*_e_ indicated the quantitative location of azide group at the chain end. These results strongly indicated the successful preparation of azide-terminated linear polymers PS-N_3_, PDMA-N_3_ and PCL-N_3_.

The dimerization of these polymers was then performed *via* self-accelerating DSPAAC. Fig. 1B–D show the GPC characterization of the resultant PS dimer (Di-PS), PDMA dimer (Di-PDMA) and PCL dimer (Di-PCL) respectively. Compared to those of the azide-terminated polymers (black curves), the GPC curves of the dimers (red curves) preserved the well-defined monomodal and symmetrical peak shape, while presented *ca.* double *M*_n_ values of their precursors for all cases. In the FT-IR spectra of the dimers (Fig. S3B–D†), the azide absorption peak at 2100 cm^−1^ disappeared completely after the dimerization for all cases. In the ^1^H-NMR spectrum of Di-PS (Fig. 2B), the peak of *H*_a_ disappeared at 3.95 ppm, compared to that of PS-N_3_ (Fig. S5B†). In the ^1^H-NMR spectra of Di-PDMA and Di-PCL (Fig. 2C and D), the peak area ratio between *H*_a_ and the protons of DIBOD clearly demonstrated the quantitative formation of dimers for both cases. These results strongly indicated the successful preparation of polymer dimers based on the combination of controlled polymerization and self-accelerating DSPAAC.

### Dimerization of 2(PCL)-N_3_

The DSPAAC based dimerization was further applied to prepare non-linear symmetric polymers. As an example, 4-arm PCL was prepared by dimerization of 2(PCL)-N_3_ with an azide pendant in the middle of the PCL chain (Scheme 2). Briefly, 2(PCL)-Br was synthesized by ROP, using a Br-functionalized dihydroxyl initiator (compound 3). Then the Br group in the middle of the chain was substituted by azide group in the presence of NaN_3_. Fig. S2D† shows the GPC curves of 2(PCL)-Br and 2(PCL)-N_3_, in which a monomodal and symmetrical peak shape was observed for both cases. The corresponding *M*_n_ and PDI were calculated as 20 700 and 1.04 for 2(PCL)-Br and 21 100 and 1.04 for 2(PCL)-N_3_, respectively. In the FT-IR spectra (Fig. S3E†), 2(PCL)-N_3_ shows the azide absorption peak at 2100 cm^−1^, compared to that of 2(PCL)-Br. Fig. S8† shows the ^1^H-NMR spectra of 2(PCL)-Br and 2(PCL)-N_3_. The peak area ratio of 4/3/2 among *H*_a_, *H*_b_ and *H*_c_ indicated the loyalty of the functional group in the middle of the chain for both cases. The peak of α-H of the terminal moved from 3.43 ppm (*H*_c_ of 2(PCL)-Br) to 3.29 ppm (*H*_C_ of 2(PCL)-N_3_) after azidation. These results confirmed the successful formation of 2(PCL)-N_3_.

**Fig. 3:**
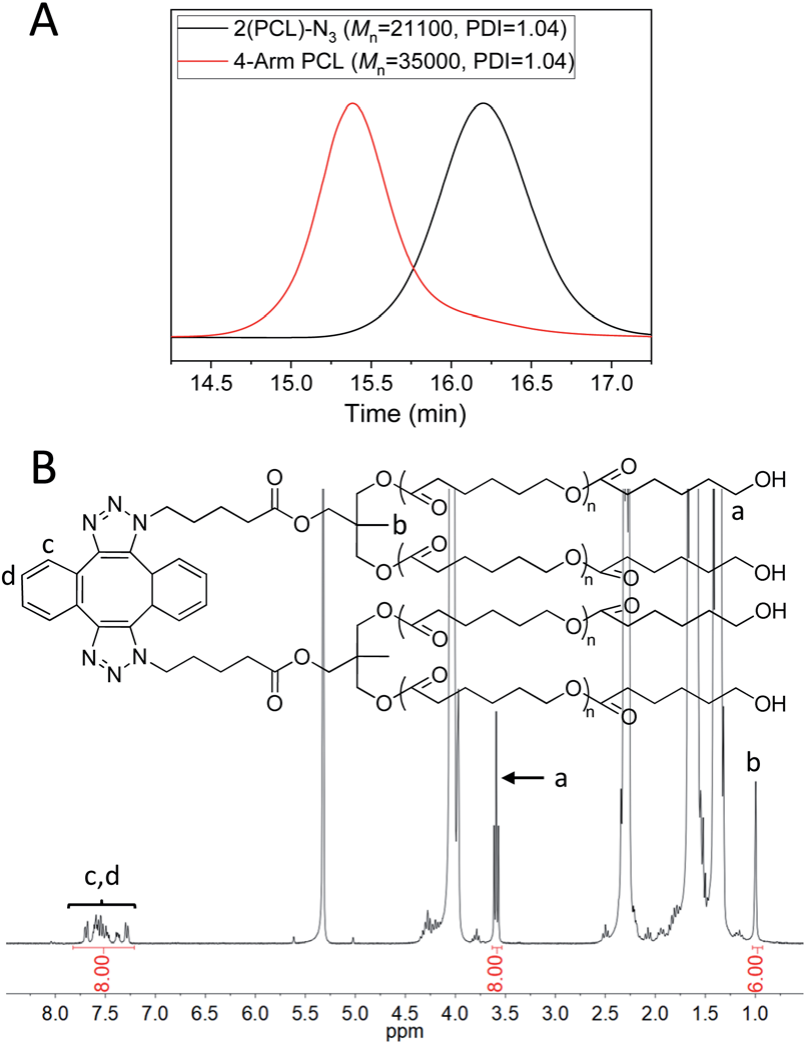
(A) GPC curves of 2(PCL)-N_3_ (black) and 4-arm-PCL (red). DMF was used as the eluent, and polystyrene standards were used for calibration. (B) ^1^H-NMR spectrum of 4-arm PCL in CD_2_Cl_2_.

4-Arm PCL was then prepared *via* the dimerization of 2(PCL)-N_3_. Fig. 3A (red) shows the GPC curve of 4-arm PCL, in which a monomodal and symmetrical peak shape was observed with PDI of 1.04. Additionally, the *M*_n_ of 4-arm PCL was calculated as 35 000, significantly smaller than double *M*_n_ of 2(PCL)-N_3_ (Fig. 3A, black, *M*_n_ = 21 100). This is because the molecular structure of 4-arm polymers is more compact than that of their linear counterparts. In the FT-IR spectrum of 4-arm PCL (Fig. S3E,† blue), the azide absorption peak at 2100 cm^−1^ disappeared completely, indicating a complete consumption of azide groups. Fig. 3B shows the ^1^H-NMR spectrum of 4-arm PCL, in which the peak area ratio of 8/6/8 among *H*_a_, *H*_b_ and *H*_c,d_ clearly demonstrated the quantitative formation of 4-arm PCL.

### Dimerization of G3-N_3_

Dimerization is the preferred method for the preparation of symmetrical dendrimers. As shown in Scheme 2, the third generation dendron G3-N_3_ was used to implement the self-accelerating DSPAAC based dimerization. Briefly, the deprotected G3-Boc reacted with compound 4 to produce G3-N_3_ through a newly formed carbamate linkage. Fig. S2E† shows the GPC curves of G3-Boc and G3-N_3_, in which the monomodal and symmetrical peak shape was observed for both cases. The corresponding *M*_n_ and PDI were 13 100 and 1.01 for G3-Boc and 13 400 and 1.01 for G3-N_3_, respectively. The FT-IR spectrum of G3-N_3_ (Fig. S3F,† red) shows the azide absorption peak at 2100 cm^−1^, compared to that of G3-Boc (Fig. S3F,† black). Fig. S9† shows the ^1^H-NMR spectrum of G3-N_3_, in which the peak area ratio of 2/16 between *H*_q_ and *H*_b_ strongly indicated the quantitative azide functionalization of G3-N_3_.

**Fig. 4:**
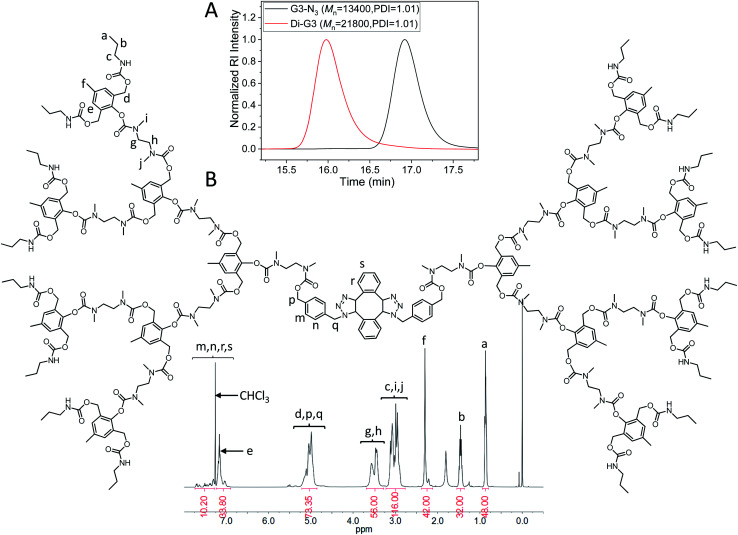
(A) GPC curves of G3-N_3_ (black) and Di-G3 (red). DMF was used as the eluent, and polystyrene standards were used for calibration. (B) ^1^H-NMR spectrum of Di-G3 in CDCl_3_.

The dimerization of G3-N_3_ was then performed using DIBOD as the small linkers. Fig. 4A (red) shows the monomodal and symmetrical GPC curve of Di-G3. The corresponding *M*_n_ and PDI were 21 800 and 1.01, respectively. The *M*_n_ value of Di-G3 was smaller than double *M*_n_ of G3-N_3_, mainly due to the compact molecular structure of the dendrimers. In the FT-IR spectra (Fig. S3F†), Di-G3 shows no azide absorption peak residue at 2100 cm^−1^, compared to that of G3-N_3_. The ^1^H-NMR spectrum of Di-G3 and the related peak assignments were shown in Fig. 4B, in which the peak area ratio of 44/32 between *H*_e,m,n,r,s_ (a total of 44H) and *H*_b_ agreed with the theoretical value. These results strongly indicated the successful formation of Di-G3.

## Conclusions

A convenient and efficient method was developed to prepare topological polymers with a symmetric molecular structure by dimerizing azide terminated polymers based on the self-accelerating DSPAAC click reaction. This is a practical method since the azide terminated polymers could be easily prepared by both post-modification and controlled polymerization techniques. The self-accelerating DSPAAC reaction was then applied to dimerize the azide terminated polymer precursors with DIBOD as small linkers. A series of topological polymers with block, 4-arm star and dendritic shapes were prepared with a symmetric molecular structure. Because of the self-accelerating property of DSPAAC, this dimerization method eliminated the requirement of equimolar amounts of the complementary reaction groups. Moreover, the usage of excess DIBOD small linkers could improve the preparation efficiency of the polymer dimers. The resultant polymer dimers could be simply purified by precipitation or dialysis to remove the excess DIBOD small linkers. Considering the distinct advantages of metal-free, mild reaction condition, and high efficiency, the DSPAAC based polymer dimerization method is expected to become a powerful tool for the formation of topological polymers with a symmetric molecular structure.

## Conflicts of interest

There are no conflicts to declare.

## Supplementary Material

RA-010-C9RA09919K-s001
