# Supplementary material for: Efficient polymer dimerization method based on self-accelerating click reaction
Source: RSC Adv. 2020 Feb 13;10(12):6794–800. doi: 10.1039/c9ra09919k (PMC9049738; doi:10.1039/c9ra09919k)
Supplement: RA-010-C9RA09919K-s001 [file RA-010-C9RA09919K-s001.pdf]

Electronic Supplementary Material (ESI) for RSC Advances.
This journal is © The Royal Society of Chemistry 2020

SUPPORTING INFORMATION

Efficient Polymer Dimerization Method Based on Self-Accelerating Click Reaction

Xueping Liu,[a,c] Ying Wu,[b] Minghui Zhang,[b] Ke Zhang*[a,c]

[a] State Key Laboratory of Polymer Physics and Chemistry, Institute of Chemistry, The Chinese Academy of Sciences, Beijing 100190, China.

[b] Institute of Polymer Chemistry and Physics, Beijing Key Laboratory of Energy Conversion and Storage Materials, College of Chemistry, Beijing Normal University, Beijing 100875, China

[c] School of Chemistry and Chemical Engineering, University of Chinese Academy of Sciences, Beijing 100049, China.

* corresponding author: kzhang@iccas.ac.cn

Experimental

Material

Monomethoxyl poly(ethylene oxide) (PEO-OH, M_n = 1900), *p*-toluenesulfonyl chloride (PTSC), triethylamine (TEA), trifluoroacetic acid (TFA), 4-nitrophenyl chloroformate, benzyl bromide, styrene (St), 4,4'-dinonyl-2,2'-bipyridine (DNBPY), 2,2'-azoisobutyronitrile (AIBN), CuBr, NaN_3, $NaHCO_3$, *N,N'*-dimethylacrylamide (DMA), 2-(hydroxymethyl)-2-methylpropane-1,3-diol, ε-caprolactone (CL), 5-bromopentanoic acid, 1,5,7-triazabicyclo[4.4.0]dec-5-ene (TBD), benzoic acid, methyl 4-bromomethylbenzoate, 1-(3-dimethylaminopropyl)-3-ethylcarbodiimide hydrochloride (EDC·HCl), 4-(dimethylamino)pyridine (DMAP), diisobutylaluminum hydride (DIBAL-H), anhydrous $MgSO_4$, methanol, petroleum ether (PE), ethyl acetate (EA), dichloromethane (DCM), diethyl ether, hexane, *N,N*-dimethylformamide (DMF), tetrahydrofuran (THF) and dimethyl sulphoxide (DMSO) were purchased as regent grade from Alfa Aesar, Aldrich, Acros, J&K Chemical, or Beijing Chemical Reagent Co. and used as received unless otherwise noted. AIBN was recrystallized from ethanol and stored at 4 °C. St and DMA were distilled under reduced pressure just prior to use. CL was dried over calcium hydride and distilled under reduced pressure just prior to use. DCM was dried over CaH_2 and distilled. Azide functionalized RAFT agent **1**, 4-(azidomethyl)benzoic anhydride,[1] *t*-butyloxy carbonyl third generation dendron (G3-Boc)[2] and DIBOD[22,263,4] were synthesized according to the previous literature.

Characterization

^{1}H-NMR spectra were recorded on a Bruker Avance 400 spectrometer at room temperature.

Fourier transform infrared (FT-IR) spectra were recorded on a Thermo Nicolet Avatar-330 Spectrometer at room temperature.

Gel permeation chromatography (GPC) was conducted on a system comprised of two Agilent PLgel columns (Mixed C and Mixed D), a Waters 1515 isocratic HPLC pump, and a Waters 2414 RI detector. DMF with 0.01 mol/L LiBr was used as the eluent at a flow rate of 1.0 mL/min. Polystyrene standards were used for calibration.

Synthesis of PEO-N_3

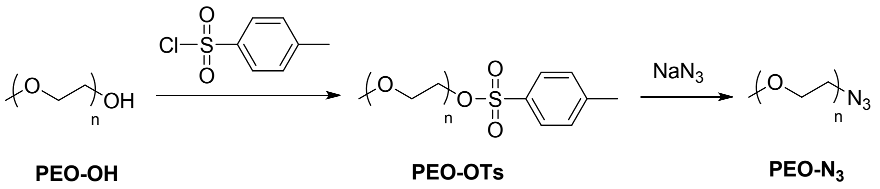

PEO-OH (15 g, 7.89 mmol), PTSC (2.54 g, 15.78 mmol) and TEA (3.8 mL. 19.73 mmol) were dissolved in DCM (40 mL). The mixture was kept stirring for 2 days at room temperature, and then passed through a short basic alumina column. After removing DCM under reduced pressure, the resultant mixture was precipitated into diethyl ether for 3 times. The precipitate was dried under vacuum to produce *p*-toluenesulfonyl-terminated PEO (PEO-OTs, 15.6 g, yield 96%). GPC determined M_n = 13400 and PDI = 1.02.

PEO-OTs (14.15 g, 6.89 mmol) and NaN_3 (4.48 g, 68.9 mmol) were dissolved in DMF (40 mL). After stirring at 50 °C for 36 h, the reaction mixture was concentrated, diluted with THF (100 mL) and passed through a short basic alumina column. After removing THF under reduced pressure, the resultant mixture was precipitated into diethyl ether. The precipitate was dried under vacuum to produce PEO-N_3 (13 g, yield 98%). GPC determined M_n = 12600 and PDI = 1.02.

Synthesis of PS-N_3

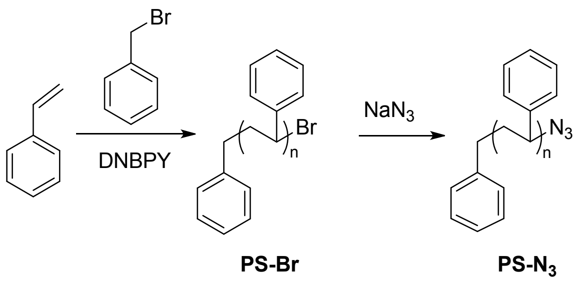

Benzyl bromide (17.8 mg, 0.25 mmol), DNBPY (0.2 g, 0.5 mmol) and St (5.2 g, 50 mmol) were mixed in a 25 mL Schlenk tube. After degassing through 4 freeze-evacuate-thaw cycles, CuBr (72 mg, 0.5 mmol) was added to the frozen mixture under N_2. After another 3 freeze-evacuate-thaw cycles, the mixture was stirred at 90 °C for 7 h and then quenched by exposure to air. The mixture was diluted with THF (50 mL), passed through a short basic alumina column, concentrated and precipitated into MeOH for 3 times. After drying under vacuum, Br-terminated PS (PS-Br, 1.56 g) was obtained with a monomer conversion of 30% from ^{1}H-NMR. Degree of polymerization (DP) of PS-Br was calculated as 60. GPC determined M_n = 16300 and PDI = 1.06.

PS-Br (1.25 g, 0.2 mmol) and NaN_3 (0.13 g, 2 mmol) were dissolved in DMF (4 mL) and kept stirring at 50 °C for 2 days. The reaction mixture was concentrated, diluted with THF (50 mL) and passed through a short basic alumina column. After removing THF under reduced pressure, the resultant mixture was precipitated into MeOH. The precipitate was dried under vacuum to produce PS-N_3 (1.23 g, yield 95%). GPC determined M_n = 16200 and PDI = 1.06.

Synthesis of PDMA-N_3

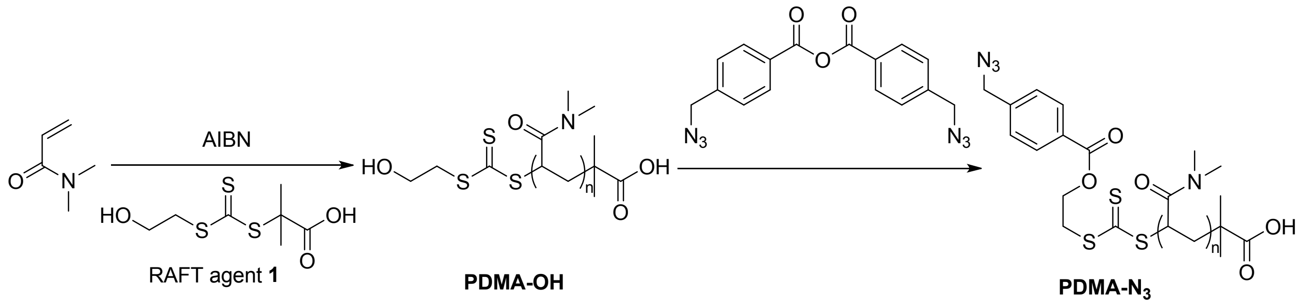

AIBN (16.4 mg, 0.1 mmol), RAFT agent **1** (0.12 g, 0.5 mmol) and DMA (4.95 g, 50 mmol) were mixed in a 25 mL Schlenk tube. After degassing through 3 freeze-evacuate-thaw cycles, the mixture was stirred at 50 °C for 1.5 h and then quenched by exposure to air. The mixture was diluted with THF (2 mL) and precipitated into diethyl ether for 3 times. After drying under vacuum, hydroxyl-terminated PDMA (PDMA-OH, 1.13 g) was obtained with a monomer conversion of 23% from ^{1}H-NMR. DP of PDMA-OH was calculated as 23. GPC determined M_n = 16500 and PDI = 1.05.

PDMA-OH (0.5 g, 0.2 mmol), 4-(azidomethyl)benzoic anhydride (0.34 g, 1 mmol) and DMAP (0.12 g, 1 mmol) were dissolved in DCM (20 mL) and kept stirring at room temperature for 2 days. The reaction mixture was concentrated, diluted with THF (2 mL) and precipitated into diethyl ether. The precipitate was dried under vacuum to produce PDMA-N_3 (0.5 g, yield 98%). GPC determined M_n = 16400 and PDI = 1.05.

Synthesis of PCL-N_3

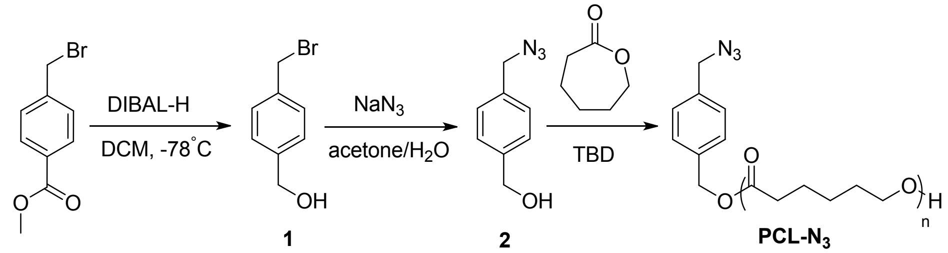

DIBAL-H (49.5 mL, 1 mol/L in THF) was added dropwise into a dry DCM solution (75 mL) of 4-bromomethylbenzoate (3.43 g, 15 mmol)

with stirring at -78 °C under N_2. The reaction was stirred at -78 °C for 1.5 h, heated up to 0 °C and quenched with H_2O. The organic layer was collected and the aqueous layer was extracted with DCM (100 mL × 2). The combined organic layer was dried over anhydrous $MgSO_4$ and concentrated to produce compound **1** as a white solid (2.95 g, yield 98%). ^{1}H-NMR (400 MHz, CD_2Cl_2, δ): 7.40 (d, 2H), 7.32 (d, 2H), 4.72 (s, 2H), 4.36 (s, 2H).

Compound **1** (2.01 g, 10 mmol), NaN_3 (975 mg, 15 mmol) were dissolved in a mixed solvent of acetone/H_2O (50 mL, v/v = 3/1) and kept stirring at 50 °C for 4 h. The organic layer was collected and the aqueous layer was extracted with DCM (50 mL × 2). The combined organic layer was dried over anhydrous $MgSO_4$ and concentrated. The crude product was purified by column chromatography on silica gel (PE/EA, v/v = 2/1) to afford compound **2** as a light yellow oil (1.27 g, yield 78%).1H-NMR (400 MHz, CD2Cl2, δ): 7.38 (d, 2H), 7.32 (d, 2H), 4.68 (s, 2H), 4.34 (s, 2H).

Compound 2 (86 mg, 0.52 mmol), TBD (18.7 mg, 0.13 mmol) and CL (3 g, 26.3 mmol) were mixed, kept stirring for 70 min and quenched by adding benzoic acid (7.3 mg, 0.65 mmol). The mixture was precipitated into a mixed solvent of diethyl ether/hexane (v/v = 2/1) for 3 times at 0 °C. After drying under vacuum, PCL-N_3 (0.9 g) was obtained with a monomer conversion of 36% from ^{1}H-NMR. DP of PCL-N_3 was calculated as 18. GPC determined M_n = 13500 and PDI = 1.05.

Synthesis of 2(PCL)-N_3

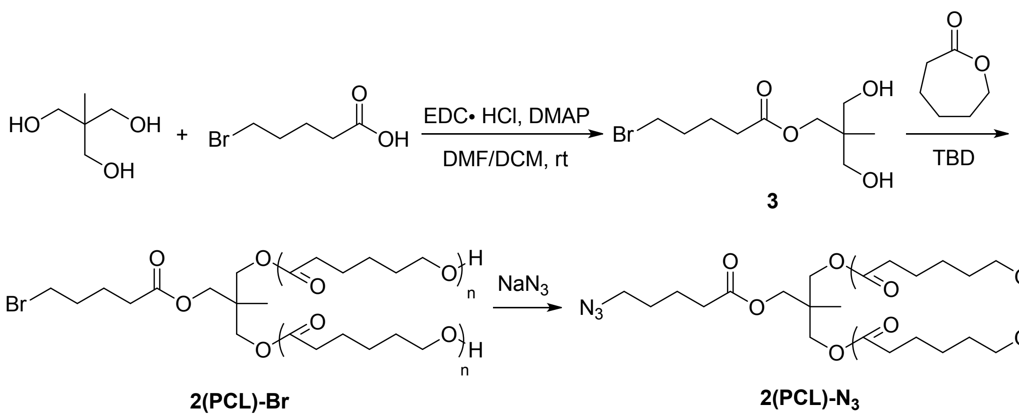

2-(Hydroxymethyl)-2-methylpropane-1,3-diol (7.21 g, 60 mmol), 5-bromopentanoic acid (2.71 g, 15 mmol) and EDC·HCl (3.46 g, 18 mmol) were dissolved in a mixed solvent of DMF/DCM (35 mL, v/v = 3/4). The reaction mixture was stirred for 6 h and then concentrated. The crude product was purified by column chromatography on silica gel (PE/EA, v/v = 2/1) to afford compound **3** as a light yellow oil (1.27 g, yield 30%). ^{1}H-NMR (400 MHz, CD_2Cl_2, δ): 4.13 (s, 2H), 3.43-3.56 (m, 6H), 2.46 (s, 2H), 2.37-2.42 (t, 2H), 1.74-1.93 (m, 4H), 0.83 (s, 3H).

Compound **3** (123 mg, 0.44 mmol), TBD (30.5 mg, 0.22 mmol) and CL (5 g, 44 mmol) were mixed, kept stirring for 10 min and quenched by adding benzoic acid (134 mg, 1.1 mmol). The mixture was precipitated into a mixed solvent of diethyl ether/hexane (v/v = 2/1) for 3 times at 0 °C. After drying under vacuum, 2(PCL)-Br (1.23 g) was obtained with a monomer conversion of 33% from ^{1}H-NMR. DP of 2(PCL)-Br was calculated as 33. GPC determined M_n = 20700 and PDI = 1.04.

2(PCL)-Br (0.5 g, 0.12 mmol) and NaN_3 (80 mg, 1.2 mmol) were dissolved in DMF (5 mL) and kept stirring at 50 °C for 2 days. The reaction mixture was concentrated, diluted with THF (50 mL) and passed through a short basic alumina column. After removing THF under reduced pressure, the resultant mixture was precipitated into a mixed solvent of diethyl ether/hexane (v/v = 2/1) at 0 °C. The precipitate was dried under vacuum to produce 2(PCL)-N_3 (0.48 g, yield 97%). GPC determined M_n = 21100 and PDI = 1.04.

Synthesis of G3-N_3

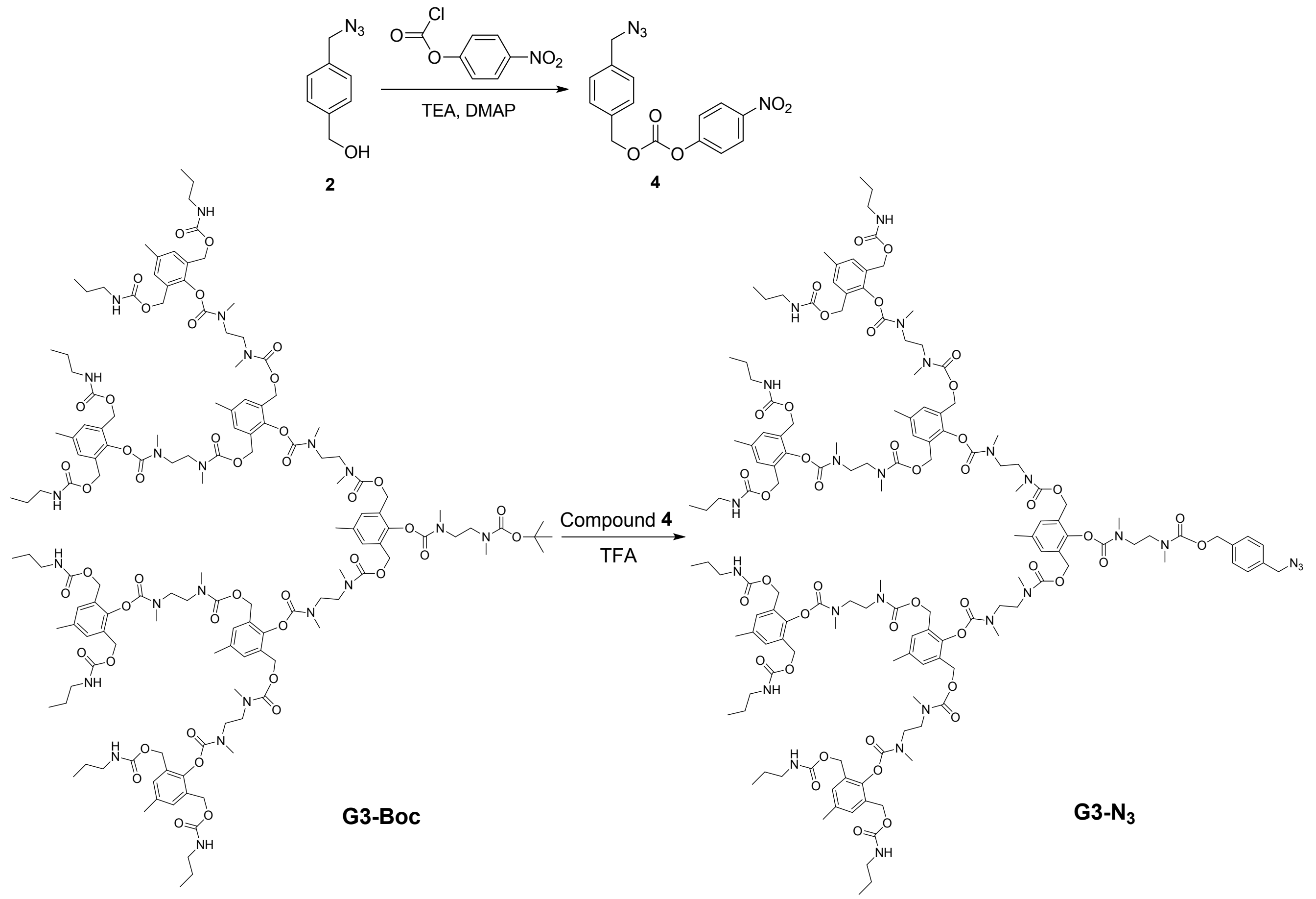

Compound **2** (375 mg, 2.3 mmol) and TEA (3 mL) in 5 mL DCM were added dropwise into a 30 mL DCM solution of 4-nitrophenyl chloroformate (0.75 g, 3.7 mmol) and DMAP (56 mg, 0.46 mmol) under stirring. The reaction mixture was stirred at room temperature for 30 min, diluted by 100 mL DCM and washed with 100 mL saturated $NaHCO_3$ aqueous solution. The organic layer was dried over anhydrous $MgSO_4$ and concentrated on a rotary evaporator. The crude product was purified by a silica gel column with EA/PE (v/v = 1/4) as the eluent to afford compound **4** as a white powder (0.53 g, yield 70%). ^{1}H-NMR (400 MHz, $CDCl_3$, δ): 8.27 (d, 2H), 7.47 (d, 2H), 7.38 (m, 4H), 5.31 (s, 2H), 4.38 (s, 2H).

G3-Boc (0.2 g, 69 μmol) was dissolved in 2 mL TFA and stirred at room temperature for 5 min. After TFA was removed under the reduced pressure, compound **4** (27 mg, 83 μmol) and TEA (2 mL) in 4 mL DMF was added

dropwise to the reaction mixture. The reaction mixture was stirred at room temperature for 10 min, diluted by 50 mL EA and washed with 50 mL saturated $NaHCO_3$ aqueous solution. The organic layer was dried over anhydrous $MgSO_4$ and concentrated on a rotary evaporator. The crude product was purified by a silica gel column with EA/methanol (v/v = 10/1) as the eluent to afford G3-N_3 as a light yellow powder (125 mg, yield 61%). GPC determined M_n = 13400 and PDI = 1.01.

Preparation of polymer dimers

The dimerization was performed in THF solution with different molar ratios of azide functionalized polymer/DIBOD, while the concentration of aizde functionalized polymers was 0.1 mol/L. A general protocol of azide functionalized polymer/DIBOD = 1/1 was as follows: Azide functionalized polymer (0.05 mmol) and DIBOD (10 mg, 0.05 mmol) were dissolved in THF (0.5 mL). The mixture was stirred for 30 min and precipitated into a poor solvent of the polymer for 3 times.

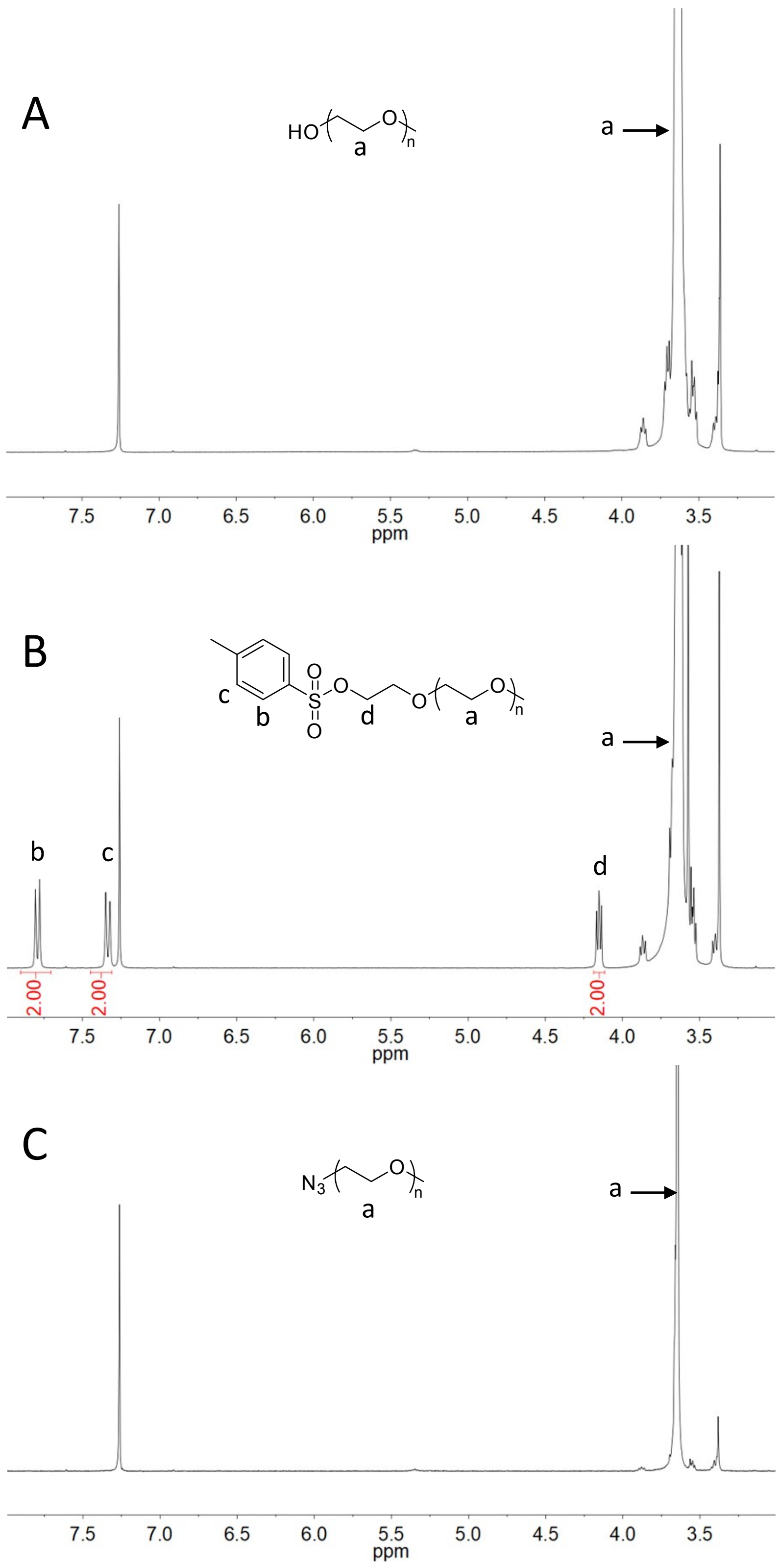

Fig. S1 ^{1}H-NMR spectra of (A) PEO-OH, (B) PEO-OTs and (C) PEO-N_3 in $CDCl_3$.

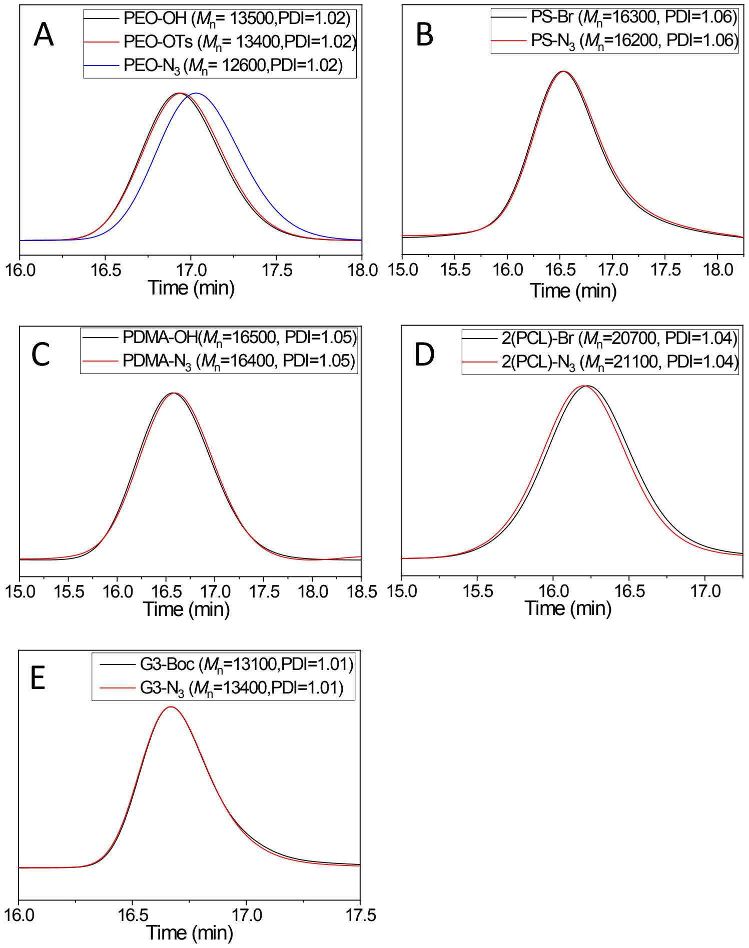

Fig. S2 GPC curves of (A) PEO-OH (black), PEO-OTs (red) and PEO-N_3 (blue); (B) PS-Br (black) and PS-N_3 (red); (C) PDMA-OH (black) and PDMA-N_3 (red); (D) 2(PCL)-Br (black) and 2(PCL)-N_3 (red); (E) G3-Boc (black) and G3-N_3 (red). DMF was used as the eluent, and polystyrene standards were used for calibration.

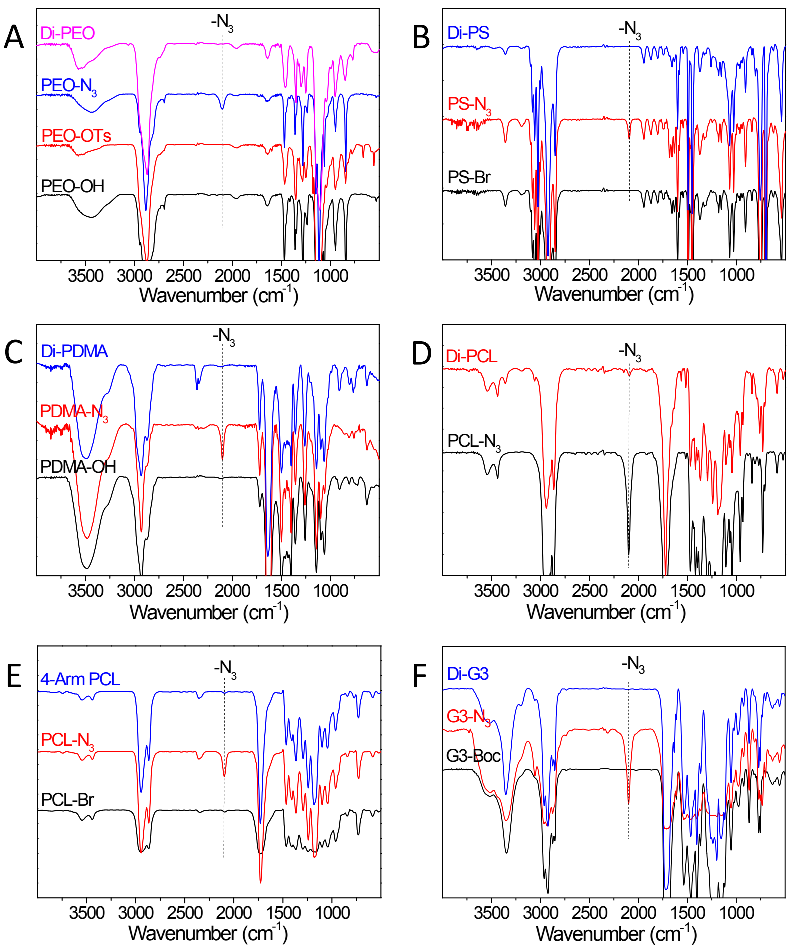

Fig. S3 FT-IR spectra of (A) PEO-OH (black), PEO-OTs (red), PEO-N_3 (blue) and Di-PEO (magenta); (B) PS-Br (black), PS-N_3 (red) and Di-PS (blue); (C) PDMA-OH (black), PDMA-N_3 (red) and Di-PDMA (blue); (D) PCL-N_3 (black) and Di-PCL (red); (E) 2(PCL)-Br (black), 2(PCL)-N_3 (red) and .4-Arm PCL; (F) G3-Boc (black), G3-N_3 (red) and Di-G3 (blue).

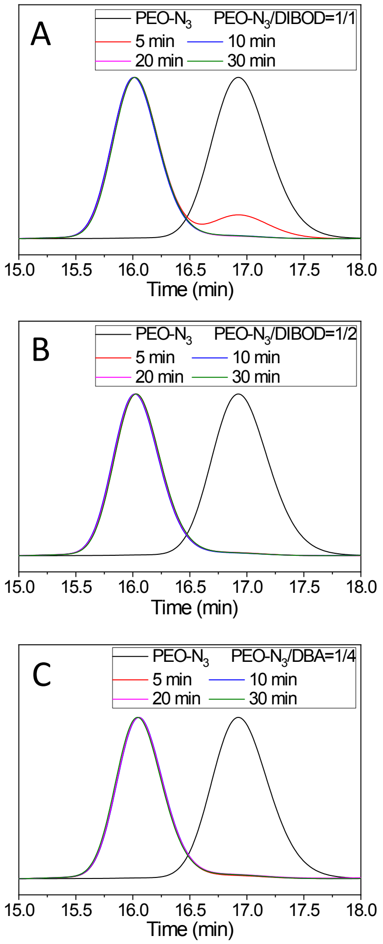

Fig. S4 GPC characterization of PEO-N_3 (black curve) and the resultant Di-PEO in different reaction times with different feed ratios between PEO-N_3 and DIBOD. Reaction time: 5 min (red), 10 min (blue), 20 min (magenta) and 30 min (olive). (A) PEO-N_3/DIBOD = 1/1, (B) PEO-N_3/DIBOD = 1/2, (C) PEO-N_3/DIBOD = 1/4. DMF was used as the eluent, and polystyrene standards were used for calibration.

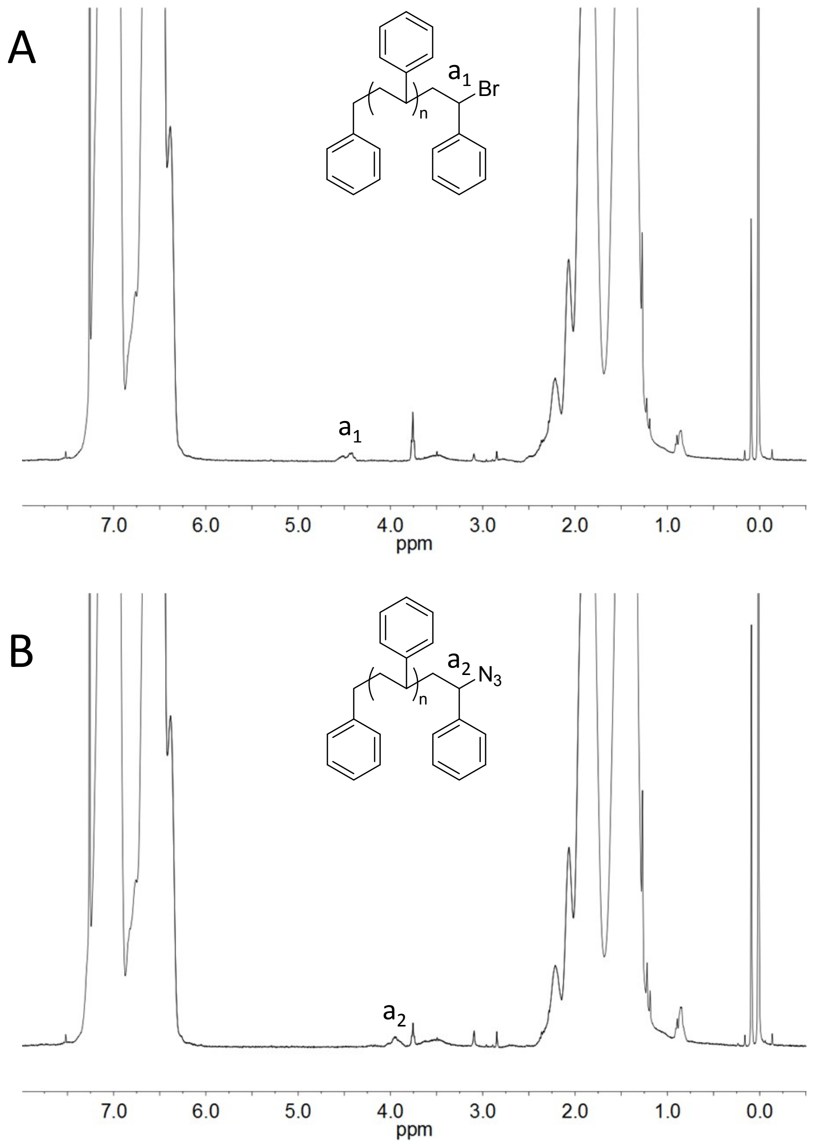

Fig. S5 ^{1}H-NMR spectra of PS-Br (A) and PS-N_3 (B) in $CDCl_3$.

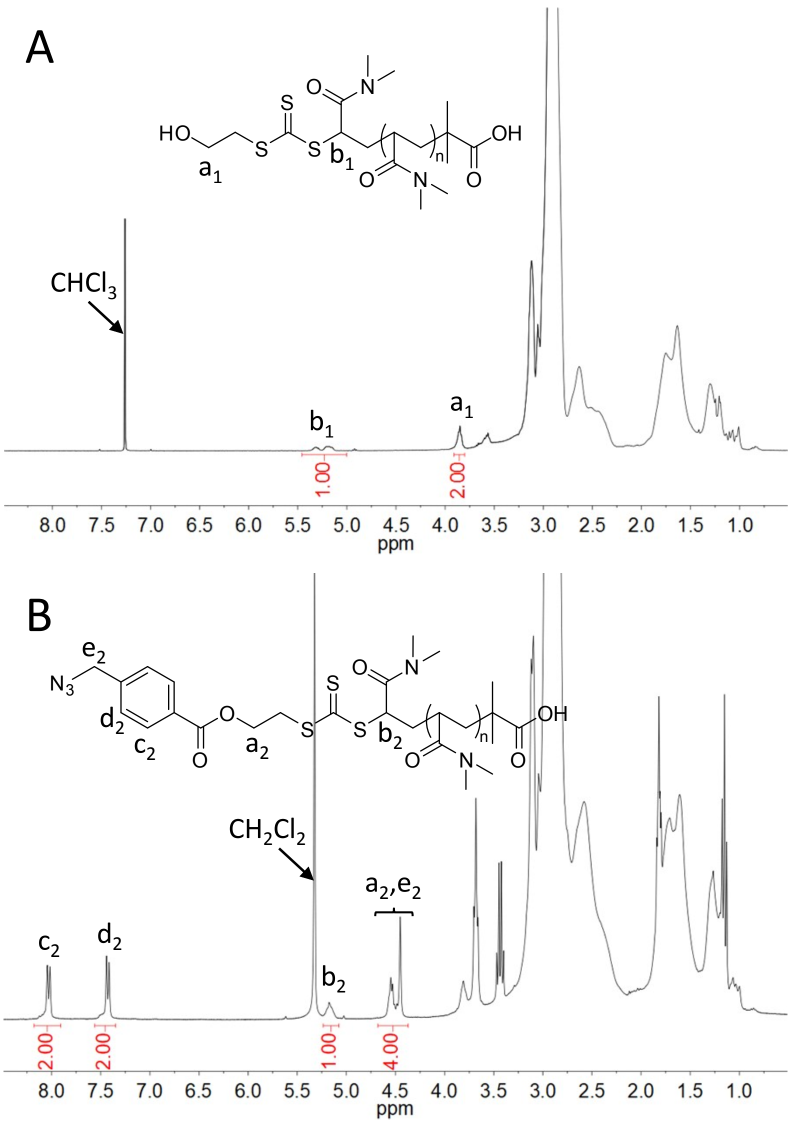

Fig. S6 ^{1}H-NMR spectra of PDMA-OH (A) in $CDCl_3$ and PDMA-N_3 (B) in CD_2Cl_2.

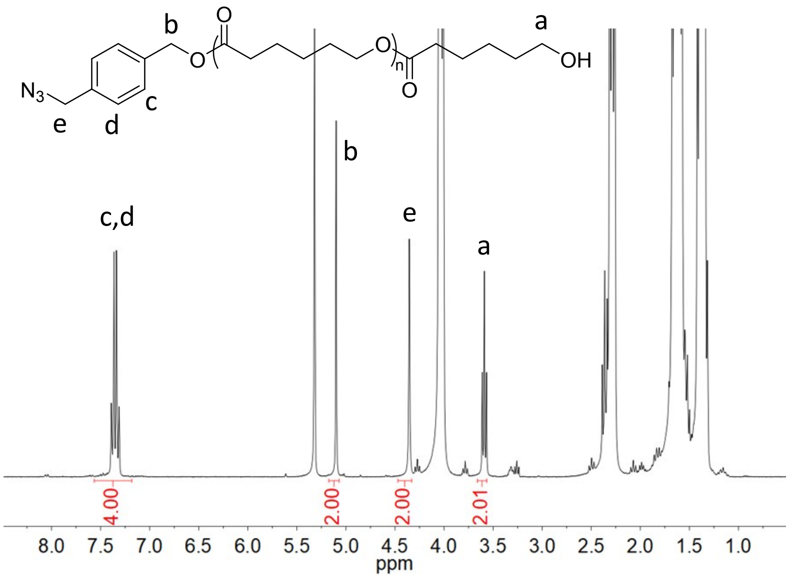

Fig. S7 ^{1}H-NMR spectrum of PCL-N_3 in CD_2Cl_2.

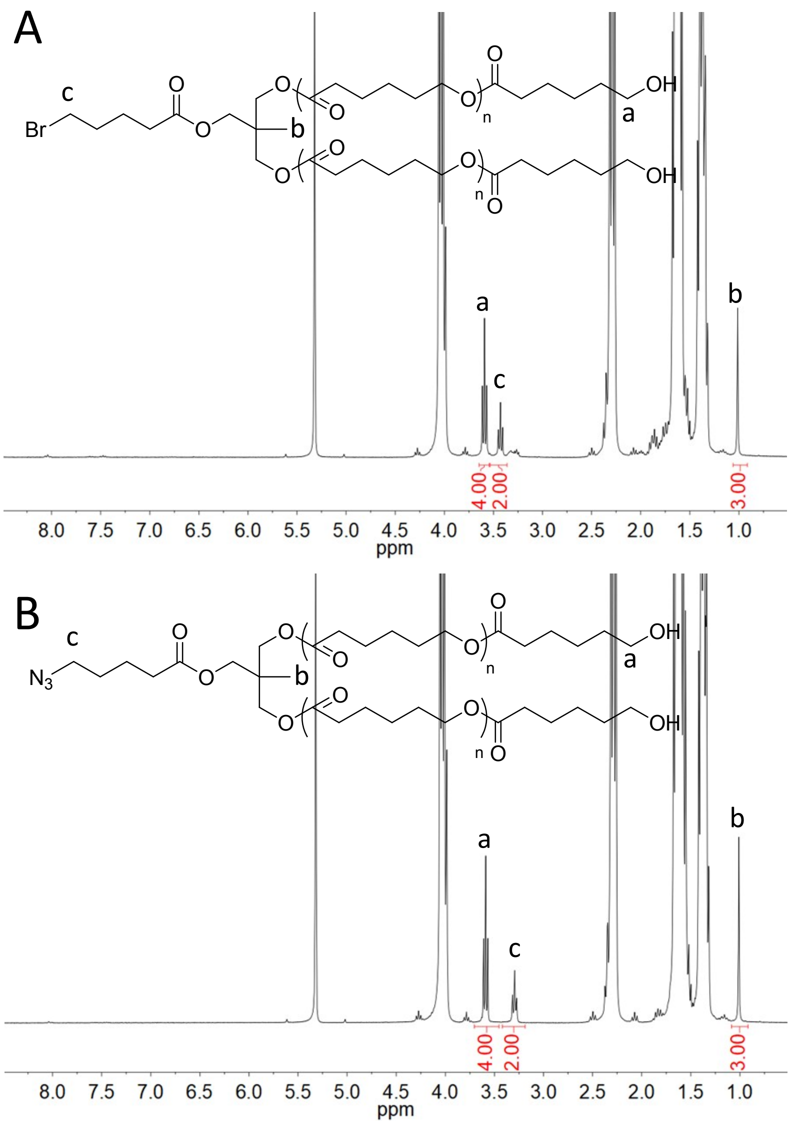

Fig. S8 ^{1}H-NMR spectra of 2(PCL)-Br (A) and 2(PCL)-N_3 (B) in CD_2Cl_2.

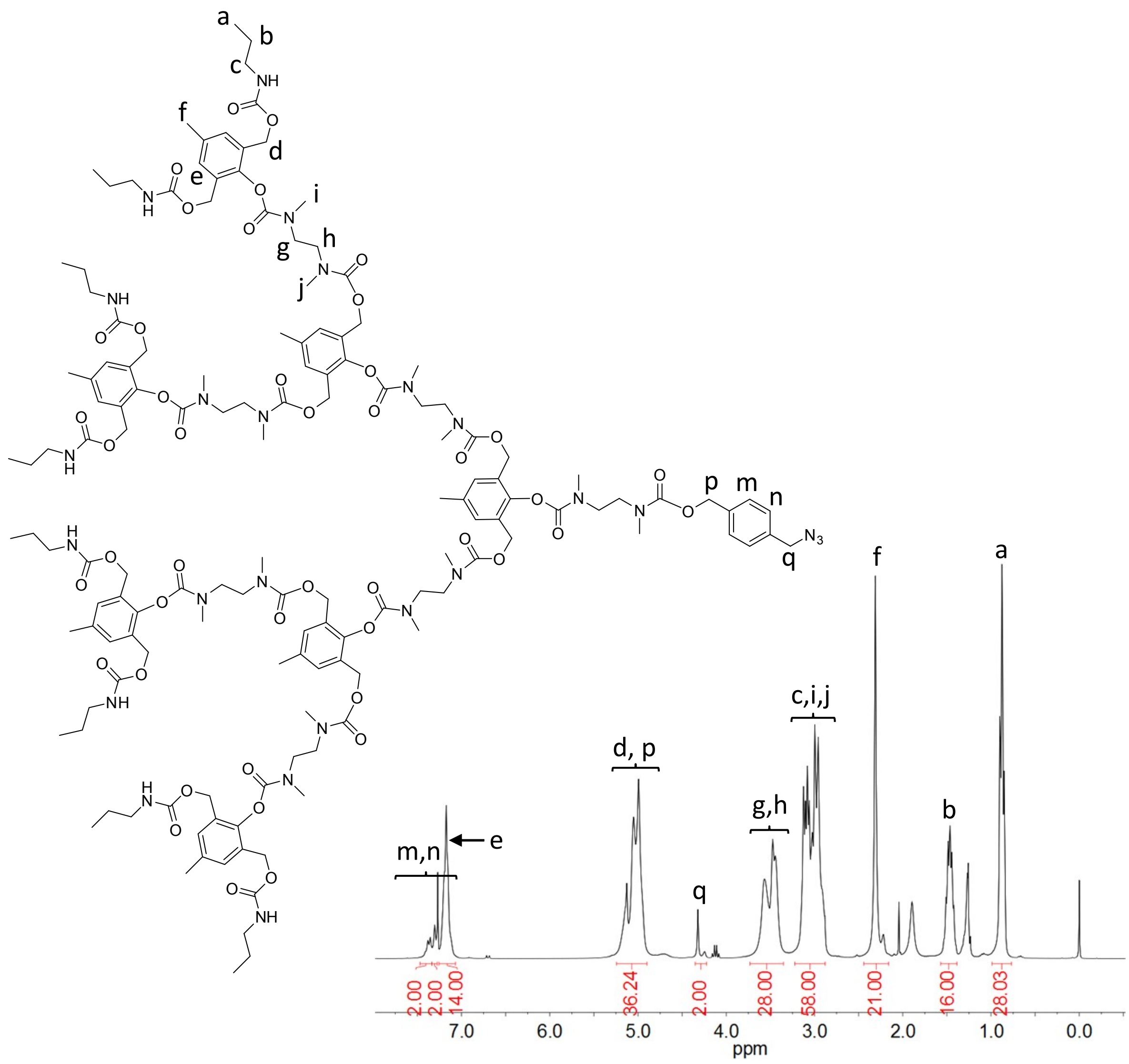

Fig. S9 ^{1}H-NMR spectrum of G3-N_3 in $CDCl_3$.

References

[1] L. Qu, P. Sun, Y. Wu, K. Zhang, Z. Liu, *Macromol. Rapid Commun.* **2017**, *38*, 1700121.

[2] R. J. Amir, N. Pessah, M. Shamis, D. Shabat, *Angew. Chem. Int. Ed.* **2003**.42, 4494.

[3] P. Sun, J. Chen, J. a. Liu, K. Zhang, *Macromolecules* **2017**, *50*, 1463.

[4] S. Chaffins, M, Brettreich, F. Wudl, Synthesis-Stuttgart 2002, 9, 1191.
